# Mr. Dawson, in Reply to Dr. Bellamy, &c.

**Published:** 1808-01

**Authors:** G. P. Dawson

**Affiliations:** Sunderland


### *To the Editors of the Medical and Physical Journal.*

Gentlemen,

THE remainder of Dr. Bellamy's Case of Diseased Bladder has recently appeared. I have read it with considerable attention. The opinion I expressed of the nature of the case, the remarks I made on the treatment of it, I thought correct and just. I think so still. It were strange indeed could I think differently. The second part of the narrative contains, like the first, obscure reasoning, extraordinary sentiments, and singular conclusions, and far from affecting the validity of my criticisms, it adds to their strength and propriety. I shall not make it the subject of particular criticism. It could answer no useful purpose, and might seem invidious. I shall content myself with making a few general observations. It is certain that Mr. Sharp, the subject of the case, had stricture in the urethra, and to it all the subsequent mischief is attributable. I make this assertion with confidence; I believe it to be true, and am convinced it is supportable by facts. Dr. Bellamy speaks incessantly of the inexplicability of Mr. Sharp's case. Where is that inexplicability to be found? Not in the case itself, but in its author's brain. To me, and and no doubt to others, the case seems perfectly clear, and attended by no circumstance not imputable to ill treated or neglected stricture. I beg leave to place Mr. Sharp's case in a perspicuous point of view. He had long laboured under stricture; no proper means were had recourse to, hence the bladder had become diseased; inflammation and suppuration took place in the perinæum and adjacent parts; mistaken, though well meant, treatment was pursued, with the injudicious and violent introduction of a catheter repeatedly; exhausted nature sunk beneath such an host of evils, general debility succeeded, gangrene followed, and death closed the scene.

The fidelity of this statement must be evident to every impartial mind. Stricture in the urethra is capable of producing inflammation, abscesses, fistulous apertures, disease of the bladder, ureters, and kidneys. No man can be ignorant of this, who is conversant with stricture, or has read the different publications on that complaint. Dr. Bellamy's idea of typhus fever, his disquisition on inflammation, his remarks on the imperfection of our art, and his speculations on other matters, cannot be read without sensations of a painful nature; and it would be wrong to rescue them, by criticism, from that oblivion, into which they will quickly and deservedly sink. I regret they were written; I am sorry they are made public. Witnessing the fate of the commencement of his narrative, it is astonishing Dr. Bellamy would publish the conclusion. It cannot do him good; it may do him harm. I admire his candour; but candour, at the expence of judgment, ceases to be a virtue.

I had intended to reply particularly to the answer, with which Dr. Bellamy has favoured my criticisms on his case. But this will not now be necessary; I shall dismiss it with a very few words. Judging by what I have said respecting abscess, Dr. Bellamy asserts my practice, in the treatment of it, must have been very confined. Were I so inclined, I might retort the assertion on him with considerable success, and more propriety. But I forbear.

He says, I advise the repeated introduction of bougies up an inflamed urethra. Here he shews the weakness of his cause, by descending to misrepresentation. I deny giving such advice. Those who have read with attention what I have written, will acquit me of the charge. He has also misrepresented my meaning on some other points; but I will not bestow on them the slightest notice. Misrepresentation is highly reprehensible; it is inimical to truth, and injures the cause it means to serve. Little

Little more remains, than for me to take my leave of Dr. Bellamy; and I proceed to do so with feelings of a very contradictory nature. I beg of him to accept my thanks, for the handsome manner in which he has received my criticisms: in taking up my pen, truth was my only object; and though I refuse to subscribe to his opinions, and believe many of them erroneous, I am impressed with a high sense of his candour and humanity.

From Dr. Bellamy, I turn to his friend Mr. Thomas. To him I have but little to say; but I hope that little will be satisfactory. When my criticisms on Dr. Bellamy's case appeared, this gentleman, stimulated by friendship, stept forward in his defence. To this I had no objection; it was what I wished; it was kind and manly. Had he stopped here, all would have been well. But he went a step further, and with asperity and flippancy adverted to a remarkable case I had written on nearly two years back; a case he never had any connection with, and one as opposite to his subject as possible. How far this was liberal and deserved from a man to whom I had given no provocation, I shall not now enquire. To the answer, which Mr. Thomas's letter impelled me to make, he has replied in a very candid and gentlemanly strain. It would be useless for me to attempt a second time to refute what he has written, inasmuch as he has nearly recapitulated all his former arguments, and to which I made an ample reply.

The whole of Dr. Bellamy's case is now before the world; so are Mr. Thomas's observations, and my criticisms. Eminent professional characters will decide on the merit of our writings, and determine which of us is right, and the most entitled to approbation. With this reflection let us be satisfied. No consideration whatever shall induce me to write another line on the case. I bid the advocates of it farewell for ever, and request them to accept my respects for the politeness and attention I have received.

I am, &c.

G. P. DAWSON.

*Sunderland, Dec. 14, 1807.*



